# A case of fulminant perimyocarditis leading to extensive ECMO treatment and spinal injury resulting in paraplegia

**DOI:** 10.1002/ccr3.1835

**Published:** 2018-11-05

**Authors:** Peter Magnusson, Charlotte Levin, Gustav Mattsson, Amanda R. Vest

**Affiliations:** ^1^ Department of Medicine, Cardiology Research Unit Karolinska Institutet Stockholm Sweden; ^2^ Centre for Research and Development Uppsala University/Region Gävleborg Gävle Sweden; ^3^ Medicinkliniken, Centralsjukhuset i Karlstad Landstinget i Värmland Karlstad Sweden; ^4^ Division of Cardiology Tufts Medical Center Boston Massachusetts

**Keywords:** cardiac arrest, cardiac resynchronization therapy, extra‐corporeal membrane oxygenation, implantable cardioverter‐defibrillator, myocarditis, perimyocarditis, spinal cord injury

## Abstract

Perimyocarditis has varying disease manifestations and prognosis. It may rapidly deteriorate into a life‐threatening state requiring advanced intensive care including veno‐arterial extra‐corporeal membrane oxygenation, which may be lifesaving. Close follow‐up is warranted to detect both short‐term and long‐term complications.

## 1 INTRODUCTION

Perimyocarditis is common and typically has a benign clinical course.^1^ However, occasionally there might be deterioration of cardiac function including arrhythmias. We present a case of fulminant perimyocarditis with cardiac arrest in a young woman who was rescued by veno‐arterial extra‐corporeal membrane oxygenation (VA‐ECMO) but suffered a prolonged complicated scenario.

## 2 CASE HISTORY

A 28‐year‐old woman with a history of mild asthma presented to the emergency room with a 2‐day history of fever (39.5°C), headache, fatigue, and chest pain. The chest pain was characterized as a dull ache with radiation to the back that worsened with inspiration.

Physical examination was initially unremarkable—she had normal peripheral perfusion, normal heart and lung auscultation, a heart rate of 71 beats per minute, and a blood pressure of 100/60 mm Hg. The first electrocardiogram showed a new left bundle branch block and a first degree atrioventricular (AV) block. The lab results showed elevated C‐reactive protein (CRP) 145 mg/L, a leukocyte count of 10.6 × 10E9/L with a peripheral eosinophilic predominance and markedly raised troponin‐T at 5423 ng/L.

Echocardiography revealed normal right‐sided chambers but regional wall motion abnormalities of the left ventricle, mainly in the apical region. No pericardial effusion or valve abnormalities were seen.

Computed tomographic angiography (CTA) ruled out thoracic aortic dissection and pulmonary embolism. The preliminary diagnosis at this point was perimyocarditis, but in anticipation of an angiography the following day, the patient still received 300 mg of acetylsalicylic acid, 180 mg of ticagrelor, and 2.5 mg of fondaparinux.

However, during transfer to the intensive care unit she developed a complete AV block with an escape rhythm of 20‐25 beats per minute. Isoproterenol infusion was initiated and an urgent angiography was performed, which showed normal coronary vessels.

After blood, nasopharynx, and urine cultures were sent, an intravenous antibiotic regimen was started with benzylpenicillin 3 g three times daily, gentamycin, and doxycycline to cover for suspected perimyocarditis due to Lyme disease.

During the following night, she developed repeated asystoles, with pauses of up to 9.5 seconds. Isoproterenol was given in incremental doses but she developed atrial flutter and an increasing number of ventricular extrasystoles. The next morning fever peaked at 39.8°C, systolic blood pressure dropped to 70‐85 mm Hg, with increasing oxygen demand, oliguria, and increased lactate and pH 7.23. Echocardiography confirmed marked systolic dysfunction with left ventricular ejection fraction (LVEF) <25% and no left ventricular chamber dilatation. It was decided to transfer the patient for treatment with VA‐ECMO at a tertiary center. She was sedated, placed on ventilator and administered dobutamine and epinephrine infusions instead of isoproterenol before the helicopter arrived.

While being loaded into the helicopter, she developed profound and extended bradycardia and circulatory collapse followed. LUCAS™ (Stryker Medical, Portage, MI, USA) Chest Compression System was started for mechanical compression during transfer, and an epinephrine infusion was given. Initial attempts to wean LUCAS™ resulted in decreasing end‐tidal CO_2_‐levels and blood pressure, but while on LUCAS™ she maintained a systolic blood pressure of 85‐100 mm Hg and oxygen saturation of 96%.

The patient was immediately cannulated onto VA‐ECMO upon arrival at the tertiary center, and a myocardial biopsy was performed. Electrical activity was present by electrocardiogram, but no mechanical systolic activity could be seen during echocardiography with biventricular myocardial standstill. A tiny pericardial effusion was present adjacent to the right ventricle, but no drainage was performed. The patient underwent targeted temperature management (TTM) to 36°C and a milrinone infusion was started, to good chronotropic and inotropic effect.

The myocardial biopsy confirmed myocarditis. Widespread infiltration of mononuclear cells could be seen but there were no giant cells nor any signs of fibrosis, metabolic disorders, or amyloidosis.

Due to her prolonged circulatory collapse, a computed tomography of brain, chest, and abdomen was performed immediately. No signs of structural damage to the brain could be seen, but signs of profound hypoperfusion were found with contrast filling defects of the liver and a collapsed aorta. During her initial VA‐ECMO treatment, laboratory results showed signs of severe ischemic damage to the liver and acute kidney injury, and she required dialysis. During the first two weeks, there was evidence of a bleeding diathesis despite administration of platelets, fresh frozen plasma, and fibrinogen, with oozing bleeds from all cannulation sites and an episode of upper gastrointestinal hemorrhage. Broad‐spectrum antibiotic treatment with meropenem was given, as was high‐dose intravenous methylprednisolone. The steroids were tapered off once the biopsy and immunological investigations resulted as negative for sarcoidosis, giant cell myocarditis, and autoimmune diseases. Despite repeated blood, urine and sputum cultures, no pathogen was isolated. Procalcitonin at arrival at the tertiary center was elevated at 0.72 μg/L, but in light of the cardiogenic shock, this is not necessarily indicative of a septic etiology.

Due to persistent complete AV block, a permanent transvenous pacemaker was placed at the end of her first week at the tertiary center. Two early but unsuccessful ECMO weaning efforts were made, and in total, she required three weeks of ECMO support before a successful wean was achieved. During the first two weeks on ECMO, her cardiac function was severely compromised with a persistently low LVEF of less than 20% and biventricular hypokinesis, and an evaluation for potential heart transplantation was begun. However, after successfully weaning, there was evidence of myocardial recovery with improved ventricular contractile function and a stable LVEF of 20%. During her fourth week at the tertiary center, she tolerated starting doses of an angiotensin‐converting enzyme inhibitor (ACEi) and a beta‐blocker, with an LVEF improvement to 30%, although with ongoing milrinone and norepinephrine support.

Unfortunately, after weaning from ECMO and sedation, the patient was discovered to be paraplegic. Magnetic resonance imaging of the spine revealed a spinal cord injury of ischemic origin with hemorrhagic transformation from the level of T6 down to the conus.

The transplantation evaluation was concluded and the patient was transferred back to the referring hospital. Inotropic agents were weaned, her ACEi and beta‐blocker doses gradually increased, and 8 weeks after her initial onset of symptoms, a cardiac resynchronization therapy defibrillator (CRT‐D) was placed. After four weeks of admission to the cardiology ward, she was transferred to the neurological ward for rehabilitation of her spinal injury. Repeated magnetic resonance imaging (MRI) showed development of atrophy of the afflicted segments of the spinal cord, indicating permanent injury. Almost two further months of rehabilitation ensued before she was discharged from the hospital. Six months later, her myocardial function had continued to recover with an LVEF of 30%‐35% by visual assessment.

## 3 DISCUSSION

This report describes a case of fulminant perimyocarditis resulting in cardiogenic shock and high‐grade AV conduction block. In response to a high pressor and inotrope requirement, the patient was successfully cardiovascularly supported on VA‐ECMO for a total of 3 weeks, with a partial recovery in biventricular myocardial function. Unfortunately, the ECMO run was complicated by a spinal cord infarction resulting in a permanent neurological deficit.

A presumptive diagnosis of acute myocarditis was based upon the acute biventricular dysfunction, fever, progressive conduction disease, development of cardiogenic shock, and troponin elevation in the absence of obstructive coronary disease. The pleuritic chest pain and pericardial effusion components suggest concurrent pericarditis. The etiology of this fulminant myocarditis episode remains elusive. High on the differential for such a presentation are giant cell, eosinophilic, lymphocytic, or sarcoid myocarditis, or alternatively an acute peripartum cardiomyopathy if within 5 months of childbirth.^1^ Given the endomyocardial biopsy findings, where giant cells and granulomas were notably absent and the infiltrate was predominantly lymphocytic, a diagnosis of giant cell, eosinophilic or sarcoid myocarditis was unlikely. An initial endomyocardial biopsy is only around 68% sensitive for giant cells and hence the pathological diagnosis of giant cell myocarditis could have been missed; however, giant cell myocarditis usually requires a 2‐3 drug immunosuppressive regimen for successful treatment, and hence recovery of electrical activity and myocardial function with only a short of course of corticosteroids would have been unlikely if giant cell myocarditis were the true diagnosis.^2^, ^3^ There were a minority of eosinophilic cells on the endomyocardial biopsy, but this is a common finding in lymphocytic myocarditis and does not indicate an eosinophilic myocarditis. However, the presence of a modest peripheral eosinophilia does maintain the possibility of a hypersensitivity myocarditis syndrome.

Lymphocytic myocarditis can be caused by a viral infection (including Coxsackie B, Coxsackie A, Echovirus, Adenovirus, Influenza, Epstein‐Barr virus, or Human Immunodeficiency Virus), bacterial infection (including Borrelia burgdorferi, Corynebacterium diphtheria, and bacterial endocarditis), autoimmune diseases (including rheumatoid arthritis, lupus and inflammatory bowel disease) and toxins (including drugs such as cocaine and cyclophosphamide).^4^ Myocardial biopsy tissue can by analyzed by viral polymerase chain reaction (PCR) to aid in the identification of an infective agent.^5^ Serologies were not drawn in this case for antibodies to Borrelia burgdorferi, the causative agent of Lyme disease, which has a relatively high incidence of 464 cases per 100 000 per year in Southern Sweden, the highest within Western Europe.^6^ No dermatological stigmata of Lyme disease were observed and there was no history of tick bites, but the presentation occurred in October, which is within the endemic Lyme season. The administration of benzylpenicillin, doxycycline, and meropenem would all have exerted anti‐bacterial activity against Borrelia.^7^

Although the etiology for this case of lymphocytic myocarditis was not determined, the underlying cause would not have significantly altered the management plan. Aside from giant cell or sarcoid myocarditis, there is no clear evidence for immunosuppressive therapy in the management of fulminant myocarditis in adults.^8^ However, the European Society of Cardiology guidelines recommend that immunosuppression may be considered on a case‐by‐case basis in lymphocytic myocarditis where no infection can be identified if the course is refractory to standard interventions.^5^

Most importantly, the myocarditis patient with refractory cardiogenic shock and rising pressor requirements should receive acute mechanical circulatory support with either VA‐ECMO as in this case, or alternatively with biventricular percutaneous axial flow catheters in cases where oxygenation is acceptable.9 Contemporary survival to discharge in patients requiring VA‐ECMO for cardiogenic shock is in the range of 50%‐60% internationally^9^; similar outcomes have been observed in patients requiring VA‐ECMO support specifically for a fulminant myocarditis indication, with 59% survival to discharge in one Japanese series.^10^ Major complications for adults supported by VA‐ECMO include hemorrhage (at cannulation sites, intrathoracic, intraabdominal, or retroperitoneal locations), arterial embolism and neurological complications, including intracerebral hemorrhage or infarction. The spinal infarct sustained in this case may have been contributed to by spinal hypoperfusion in the setting of hypotension and/or vasopressor use, or by peripheral arterial embolism to a spinal artery from the ECMO circuit. The requirement for systemic anticoagulation during ECMO support is a strong risk factor for hemorrhagic conversion of such neurological injuries.

Despite the neurological injury, substantial cardiovascular progress was achieved, with partial myocardial recovery occurring in the weeks and months after the acute myocarditis presentation. Recent data suggest that myocardial recovery is most likely in patients with non‐dilated left ventricles, the absence of an intraventricular conduction delay and a higher cardiac index at presentation.^11^ There is also a role for cardiac MRI with late gadolinium enhancement (LGE) after acute myocarditis, with the presence of LGE being a strong independent predictor of adverse clinical outcomes.^12^

In summary, this case highlights the importance of rapid access to VA‐ECMO support for patients with acute deterioration into cardiogenic shock, as occurred in this young woman with fulminant lymphocytic myocarditis. Although the risk of mortality remains high despite timely initiation of appropriate mechanical support, survival is achievable even in cases with profound conduction disease and myocardial standstill.

## CONFLICT OF INTEREST

None declared.

## AUTHOR CONTRIBUTION

PM: involved in idea, design, major writing, and project management. CL: involved in case selection, data collection, and writing. GM: performed writing. AV: performed major writing. All authors read and approved the final version of this case report for submission to the Clinical Case Report.
